# Bitter melon extract ameliorates palmitate-induced apoptosis via inhibition of endoplasmic reticulum stress in HepG2 cells and high-fat/high-fructose-diet-induced fatty liver

**DOI:** 10.29219/fnr.v62.1319

**Published:** 2018-03-22

**Authors:** Hwa Joung Lee, Rihua Cui, Sung-E Choi, Ja Young Jeon, Hae Jin Kim, Tae Ho Kim, Yup Kang, Kwan-Woo Lee

**Affiliations:** 1 Department of Endocrinology and Metabolism, Ajou University School of Medicine, Suwon, Republic of Korea; 2 Department of Physiology, Ajou University School of Medicine, Suwon, Republic of Korea; 3 Division of Endocrine and Metabolism, Department of Internal Medicine, Seoul Medical Center, Seoul, Republic of Korea

**Keywords:** bitter melon extract, palmitate, high-fat/high-fructose diet, nonalcoholic fatty liver disease, endoplasmic reticulum stress, apoptosis

## Abstract

**Background:**

Bitter melon (BM) improves glucose level, lipid homeostasis, and insulin resistance *in vivo*. However, the preventive mechanism of BM in nonalcoholic fatty liver disease (NAFLD) has not been elucidated yet.

**Aim & Design:**

To determine the protective mechanism of bitter melon extract (BME), we performed experiments *in vitro* and *in vivo*. BME were treated palmitate (PA)-administrated HepG2 cells. C57BL/6J mice were divided into two groups: high-fat/high-fructose (HF/HFr) without or with BME supplementation (100 mg/kg body weight). Endoplasmic reticulum (ER) stress, apoptosis, and biochemical markers were then examined by western blot and real-time PCR analyses.

**Results:**

BME significantly decreased expression levels of ER-stress markers (including phospho-eIF2α, CHOP, and phospho-JNK [Jun N-terminal kinases]) in PA-treated HepG2 cells. BME also significantly decreased the activity of cleaved caspase-3 (a well known apoptotic-induced molecule) and DNA fragmentation. The effect of BME on ER stress–mediated apoptosis *in vitro* was similarly observed in HF/HFr-fed mice *in vivo*. BME significantly reduced HF/HFr-induced hepatic triglyceride (TG) and serum alanine aminotransferase (ALT) as markers of hepatic damage in mice. In addition, BME ameliorated HF/HFr-induced serum TG and serum-free fatty acids.

**Conclusion:**

These data indicate that BME has protective effects against ER stress mediated apoptosis in HepG2 cells as well as in HF/HFr-induced fatty liver of mouse. Therefore, BME might be useful for preventing and treating NAFLD.

In recent years, nonalcoholic fatty liver disease (NAFLD) is commonly found in many people worldwide (1). NAFLD ranges from steatosis to nonalcoholic steatohepatitis (NASH) that can lead to hepatocellular carcinoma and liver failure (2). Obesity-related mortality, type 2 diabetes, and cardiovascular risk are particularly high in patients with NAFLD (3). Liver steatosis, a typical feature of NAFLD, appears when the sum of *de novo* fatty acid synthesis and free fatty acids (FFA) imported into liver is greater than the sum of FFA oxidation and FFA exported from the liver. Therefore, an increment in FFA level in the liver may play a key role in the development of NAFLD. Excess FFA evokes endoplasmic reticulum (ER) stress and leads to ER dysfunction (4).

Sustained exposure to FFA induces excessive protein synthesis that exceeds ER capacity, resulting in the accumulation of unfolded or misfolded proteins in the ER rumen. To solve this problem, unfolded protein response (UPR) is induced to maintain ER homeostasis. Binding immunoglobulin protein (BiP) is a member of the heat-shock protein-70 (HSP70) family protein chaperone. It is associated with ER-stress sensors such as inositol requiring enzyme-1 (IRE1), Protein kinase ribonucleic acid-like endoplasmic reticulum kinase (PERK), and activating transcription factor 6 (ATF6) in the ER membrane to detect unfolded or misfolded proteins and move them from membrane to ER rumen for repairing (5). When ER-stress sensors are dissociated from BiP, they are activated to participate in three signaling pathways of ER stress. The activation degree of IRE1, PERK, and ATF6 under UPR determines cell fate such as cell survival and cell death (6). Persistent and intense ER stress will cause ER dysfunction, eventually leading to apoptosis. Expression of C/EBP homologous protein (CHOP), a typical ER-stress marker regulated by UPR, plays a key role in inducing the apoptotic process. Expression of CHOP is known to be regulated by IRE1, PERK, and ATF6 pathways. The PERK-eIF2α-ATF4 pathway has been reported to be the most important for the expression of CHOP (7). DeZwaan et al. have reported that expression of CHOP can accelerate cell death in a variety of chronic liver diseases (8). In addition, caspase-12, an apoptotic regulator, is activated by the IRE-TRAFT2-JNK pathway. It then induces apoptosis by sequentially activating caspase-9 and effecter caspase-3 (9). Strong ER stress induces nuclear condensation, DNA fragmentation, and formation of apoptotic bodies containing cleaved DNA and proteolytic fragments. Since apoptosis is associated with ER-stress seen in the progression of NAFLD and is positively correlated with disease severity, and it can be used as a disease progression marker (7).

Bitter melon (BM) belongs to the family of Cucurbitaceae. It is cultivated in tropical regions of Asia, Africa, and South America. BM has long been used as traditional medicines in Asia. It is widely known to have antidiabetic, antioxidant, antiviral, and anticancer effects. The efficacy of BM for several diseases has been proven. It has been reported that bitter melon extract (BME) can reduce the expression of ER-stress proteins such as ATF6, XBP1, PERK, and CHOP in colonic epithelial cells. BME is also known for improving high fat diet (HFD)-induced obesity and hyperlipidemia in animal models (10, 11). Beneficial effects of BME on obesity and hyperlipidemia in liver and skeletal muscle are regulated by fat metabolizing kinases and nuclear factors (12). The liver protective effect of BME is mainly due to its antioxidant effects by scavenging free radicals and reducing inflammation caused by harmful stimuli (13).

Although several possible mechanisms have been proposed for the hepatoprotective effect of BME, few studies have examined the effect of BME on ER stress–mediated apoptosis in NAFLD. Therefore, the objective of the present study was to determine the effect of BME on palmitate (PA)-treated HepG2 cells (*in vitro*) and a mouse model of NAFLD (*in vivo*).

## Materials and methods

### Materials

PA and 4,6-diamidino-2-phenylindole diHCl (DAPI) were purchased from Sigma-Aldrich (St. Louis, MO, USA). Anti-phospho-Akt (#9271), Anti-Akt (#9272), Anti-caspase-3 (#9962), anti-cleaved caspase-3 (#9961), anti-calnexin (#2679), anti-CHOP (#2895), anti-phospho-c-Jun (#9164), anti-phospho-eIF2α (#9721), anti-eIF2α (#9722), anti-IκBα (#9242), anti-phospho-JNK (#9251), anti-JNK (#9252), anti-phospho-NFκB (#3031), anti-NFκB (#4764), and anti-PARP (#9542) antibodies were obtained from Cell Signaling Technology (Danvers, MA, USA). Anti-actin (sc1616) and anti-c-Jun (sc1694) antibodies were purchased from Santa Cruz Biotechnology (Santa Cruz, CA, USA).

### Cells and culture

Commercially available HepG2 human hepatoma cell line was purchased from the American Type Culture Collection (ATCC, Manassas, VA). HepG2 cells were seeded into 12-well plates, at a density of 1 × 10^5^ cells per well, and then maintained in Dulbecco’s modified Eagle’s medium (DMEM) supplemented with 10% fetal bovine serum (FBS; Gibco, Grand Island, NY, USA), 100 U/mL  penicillin, and 100 g/mL streptomycin at 37°C, in a humidified atmosphere containing 95% air and 5% CO_2_. After 24 h of incubation, HepG2 cells were treated with different concentrations of BME and 300 μM PA. Cells were treated with Dimethyl sulfoxide (DMSO) which was used as a vehicle control. PA and BME were treated for 12 h and subsequently, the cells were harvested using trypsin and washed with phosphate-buffered saline (PBS). The harvested HepG2 cells were suspended in Radioimmunoprecipitation assay (RIPA) buffer for western blotting or homogenized in RNAiso Plus reagent for real-time polymerase chain reaction (PCR).

### Preparation of PA

PA/bovine serum albumin (BSA) conjugate was prepared by saponifying PA with sodium hydroxide followed by mixing with BSA. PA (20 mM in 0.01 M NaOH) was incubated at 70°C for 30 min (14). Fatty acid soaps were then complexed with 5% fatty acid-free BSA in PBS at a 1:3 volume ratio. Complexed fatty acids consisted of 5 mM PA and 3.75% BSA. PA/BSA conjugate was then diluted in DMEM containing 10% FBS and administered to cell cultures.

### Preparation of BME

BM (*Momordica charantia*) was purchased at the BM farm located in Cheorwon-gun, South Korea. After washing it thoroughly, the fruit of BM was cut and dried naturally at room temperature; it was powdered using an electric grinder and stored at 4°C. BME was prepared from 10 g of dried BM powder using 100% ethanol. Extract was filtered and dried at 37°C for 24 h. Dried BME was dissolved in dimethyl sulfoxide (Supplementary Fig. 1). BME was stored in airtight containers at −20°C until use.

### Animal experiments

C57BL/6J male mice were purchased from Japan SLC, Inc. (Hamamatsu, Japan). All animal experiments were approved by the Animal Ethics Committee of the Laboratory Animal Research Center, Ajou University Medical Center, Suwon, South Korea. These mice were housed in a temperature controlled room (22 ± 2°C) with a 12/12 h light/dark cycle and fed *ad libitum*. To prepare the dietary NAFLD mouse model, 8-week-old male C57BL/6J mice were fed HFD (pellet containing 60% kcal fat; Research Diets, New Brunswick, USA, Cat#12492) and drinking water containing 30% high fructose (HFr) for 7 weeks. Then, these mice were randomly divided into two groups: (1) high-fat/high-fructose (HF/HFr) group (*n* = 6) and (2) HF/HFr + 100 mg/kg BME (HF/HFr/BME) group (*n* = 6). Each group was orally administered either DMSO or BME (100 mg/kg) on every other day for 5 weeks under the HF/HFr diet. The body weight was measured before the sacrifice. For glucose tolerance tests (GTT), mice were fasted for 6 h and intraperitoneally injected with glucose solution (1 g/kg; Sigma-Aldrich) or insulin (1 unit/kg; Sigma-Aldrich). Blood samples were taken at different time points (0, 15, 60, and 120 min after insulin or glucose loading) from the tail vein. Plasma glucose levels were measured using an Accu-Chek glucometer (Roche, Basel, Switzerland). After GTT measurements, mice were sacrificed using carbon dioxide. Epididymal fat, perirenal fat, subcutaneous fat, and the liver of mice were removed and weighed. The liver was suspended in RIPA buffer for western blotting or homogenized in RNAiso Plus reagent for real-time PCR.

### Measurement of triglyceride

Tissue triglyceride was extracted by using Folch extraction method and measured with commercially available kits (LabAssay^TM^ triglyceride, Wako Diagnostics, Japan) using N-ethyl-N-(2-hydroxy-3-sulfopropyl)-3,5- dimethoxyaniline sodium salt (DAOS) as a blue pigment. For biochemical analysis of blood samples, blood was obtained from 6-h-fasted mouse and immediately centrifuged at 13,000 rpm for 1 min at 4°C. The upper plasma was then collected and stored at −80°C. Plasma glucose levels were measured using the glucose oxidase method. Plasma levels of total cholesterol, triglyceride, and alanine aminotransferase (ALT) were measured using an autochemical analyzer (Cobas c111, Roche, Germany).

### Hematoxylin and eosin staining

The liver of each mouse was removed, fixed in 10% neutral-buffered formalin, embedded in paraffin, and sectioned to a thickness of 3μm. Liver sections were then stained with hematoxylin and eosin (H&E).

### Western blot analysis

RIPA buffer (150 mM NaCl, 1% NP-40, 0.5% deoxycholate, 0.1% sodium dodecyl sulfate [SDS], 50 mM Tris-HCl pH 7.5, protease inhibitor cocktail; Roche Applied Science, Mannheim, Germany) was used to extract cellular proteins. Equivalent amounts of protein (10 μg each) in SDS sample buffer (50 mM Tris-HCl pH 6.8, 2% SDS, 100 mM DL-dithiothreitol, 10% glycerol) were separated by 8−15% SDS-polyacrylamide gel electrophoresis (SDS-PAGE) and then transferred to polyvinylidene difluoride (PVDF) membranes (Millipore, Bedford, MA, USA). Target antigens were reacted with primary antibodies after blocking membranes with 5% skim milk for 30 min. After binding with secondary antibodies (horseradish peroxidase [HRP]-conjugated anti-goat Immunoglobulin G (IgG), anti-mouse IgG, or anti-rabbit IgG), immunoreactive bands were detected using an enhanced chemiluminescence system (Pierce, Rockford, IL, USA). Band intensity was determined by densitometric analysis using Quantity One D image analysis system (Bio-Rad, Hercules, CA, USA).

### DNA fragmentation assay

Cell death was determined by measuring fragmented DNA using Cell Death Detection enzyme-linked immunosorbent assay (ELISA) kit (Roche Applied Science, Mannheim, Germany). Briefly, cells were lysed by adding cell lysis buffer supplied with the kit. After centrifugation (200 × *g*, 10 min), the supernatant was pipetted onto an anti-streptavidin-coated microplate. Anti-DNA monoclonal antibody conjugated with peroxidase (anti-DNA-POD) and anti-histone-biotin were added. After incubating at 25°C for 90 min, wells were rinsed with incubation buffer (supplied by the kit) three times. The color was developed by adding 2,20-azino-di-[3-ethylbenzthiazoline sulfonate] (ABTS) substrate solution and incubated at room temperature for 10−20 min with shaking (250 rpm). The amount of peroxidase retained in the nucleosome complex was determined by measuring absorbance at a wavelength of 405 nm.

### DAPI staining

For nuclear staining, photosensitized cells were fixed with 4% paraformaldehyde (pH 7.4) for 10 min and then incubated with DAPI (1 μg/mL; Sigma-Aldrich) at 37°C for 10 min. After washing with PBS, stained cells were immediately observed under a fluorescence microscope (340 nm excitation and 388 nm emission). Cells exhibiting reduced nuclear size, chromatin condensation, intense fluorescence, and nuclear fragmentation were considered apoptotic.

### Flow cytometric measurement of reactive oxygen species

HepG2 cells grown on a 12-well plate were treated with 300 μM PA and various concentrations of BME at the same time for 4 h. Cells were detached from culture plates by digesting with trypsin (Invitrogen, Carlsbad, CA, USA) and then sedimented by centrifugation at 500 × *g* for 5 min. To quantitatively analyze reactive oxygen species (ROS), cells were immediately incubated with dichlorofluorescein diacetate (DCFDA; Sigma-Aldrich) at 37°C for 10 min. After washing twice with PBS, fluorescence intensity of stained cells was analyzed on a FACSVantage SE cytometer (BD Biosciences, San Jose, CA, USA).

### RNA isolation and quantitative real-time PCR

Total RNA was isolated from mouse liver tissues using RNAiso Plus reagent (TaKaRa Bio Inc., Tokyo, Japan) according to the manufacturer’s instructions. Then, cDNA was synthesized from total RNA using cDNA synthesis kit and used as template in PCR using gene-specific primers for interleukin (IL)-6 [CCA TCC AGT TGC CTT GGG (F) and GCC GTG GTT GTC ACC AGC AT (R)], IL-1β [TCT CGC AGC ACA TCA ACA (F) and CCT GGA AGG TCC ACG GGA AA (R)], and monocyte chemoattractant protein (MCP)-1 [CAG CCA GAT GCA GTT AAC GC (F) and GCC TAC TCA TTG GGA TCA TCT (R)]. Quantitative real-time PCR (qPCR) was performed using SYBR Green (TaKaRa Bio Inc.) on a TaKaRa TP-815 instrument. All expression values were normalized to levels of 36B4, an internal control.

### Terminal deoxynucleotidyl transferase dUTP nick end labeling assay

Deparaffinized tissue sections were treated with proteinase K (20 μg/mL) for 15 min. Endogenous peroxidase was then blocked using 3% hydrogen peroxide in PBS for 10 min. Samples were then washed three times in distilled water and incubated with terminal deoxynucleotidyl transferase buffer at room temperature for 10 min. Excess buffer was drained and samples were incubated with terminal transferase and biotin-16-dUTP at 37°C for 1 h. Samples were then rinsed four times with PBS and incubated with a 1:400 dilution of peroxidase-conjugated streptavidin at 37°C for 1 h. Slides were rinsed with PBS and incubated with 3,3-diaminobenzidine at room temperature for 5 min. Sections were then washed with PBS three times and counterstained with methyl green.

### Statistical analysis

Data are presented as means ± standard errors of the mean (SEMs) from at least three independent experiments. Statistical differences between groups were determined using Student’s *t*-test and Fisher’s exact test. *P*-values of less than 0.05 were considered statistically significant.

## Results

### BME inhibits PA-induced apoptosis in HepG2 cells

**Fig. 1:**
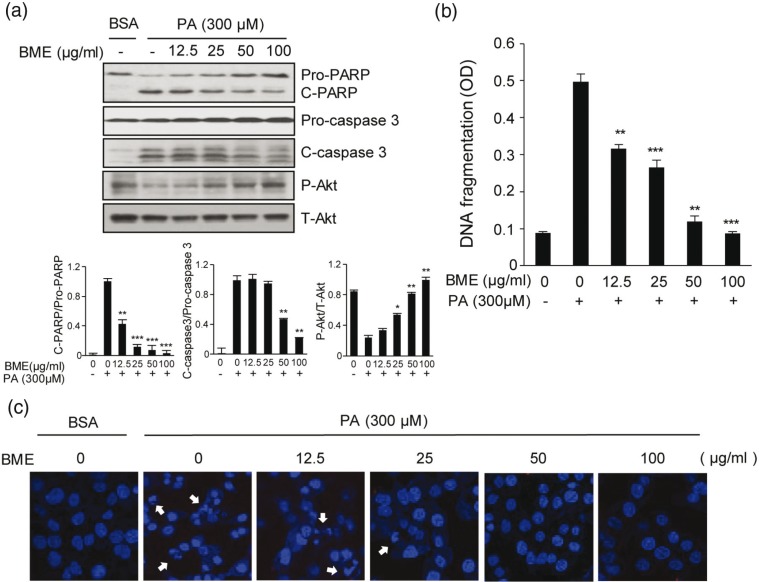
Protective effects of BME on PA-induced HepG2 cell death. HepG2 cells were treated with different concentrations of BME and 300 μM PA. DMSO was used as a vehicle. (a) HepG2 cells were collected at 12 h after treatment. Whole protein lysates were prepared using RIPA extraction buffer. PARP, cleaved caspase-3, and Akt were analyzed by western blot using anti-PARP, anti-cleaved caspase-3, and anti-Akt antibodies. Data are expressed as mean ± SD of four independent experiments. *p < 0.05, **p < 0.01, ***p < 0.001 versus PA-treated group. (b) HepG2 cells were treated with different concentrations of BME and 300 μM PA for 12 h. Fragmented DNAs were then measured using a Cell Death Detection ELISA kit. Data are expressed as mean ± SD of four independent experiments. **p < 0.01, ***p < 0.001 versus PA-treated group. (c) Nuclear DNAs in BME-treated and PA-treated HepG2 cells were stained using DAPI and observed under a fluorescence microscope (388 nm emission). Arrows indicate condensed and fragmented nucleosomes.

As a FFA, PA is cytotoxic to HepG2 cells. It can induce apoptotic cell death (15). To examine the protective effect of BME against PA-induced apoptosis, HepG2 cells were treated with 300 μM PA and different concentrations of BME. PA increased apoptosis marker such as cleaved caspase-3 (C-caspase-3) and cleaved PARP (C-PARP). However, it was noted that PA decreased the survival marker, P-Akt. Interestingly, it was noted that BME significantly prevented PA-induced C-PARP and C-caspase-3 in a dose-dependent manner. We also found that P-Akt gradually recovered as the BME concentration increased (Fig. 1a). We further investigated the effect of BME on PA-induced DNA fragmentation and nuclear condensation in HepG2 cells. Treatment with 300 μM PA increased DNA fragmentation by fivefold. However, such an increase in DNA fragmentation was significantly and dose-dependently diminished by BME (Fig. 1b). Furthermore, in PA-treated HepG2 cells, DAPI staining demonstrated nuclear condensation, a typical characteristic of apoptosis. However, BME dose-dependently blocked PA-induced nuclear condensation, with a maximum effect observed at higher concentrations of BME (Fig. 1c). Taken together, these data demonstrated that PA-induced apoptosis in HepG2 cells and that BME definitely could inhibit such apoptotic effect of PA.

### BME reduces PA-induced ER stress in HepG2 cells

**Fig. 2:**
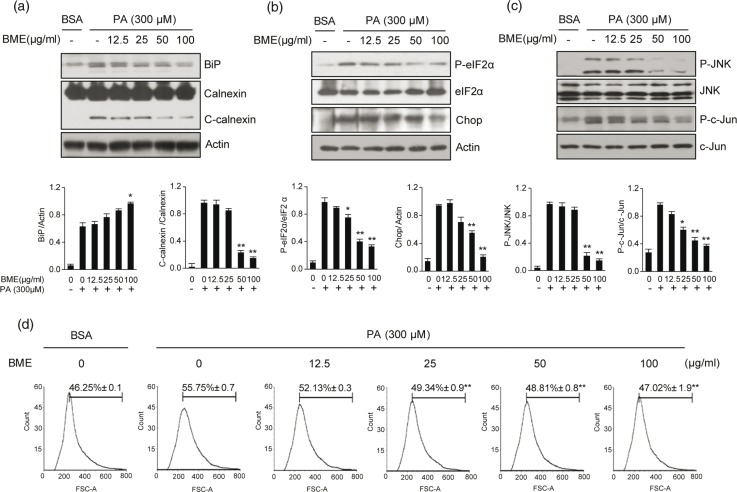
Reduction of ER stress–related markers in BME-treated HepG2 cells. HepG2 cells were treated with different concentrations of BME and 300 μM PA for 12 h. DMSO was used as a vehicle. ER stress was analyzed by measuring levels of BiP, calnexin (a), P-eIF2α, CHOP (b), P-JNK and P-c-Jun (c). Data are expressed as mean ± SD of three independent experiments. *p < 0.05, **p < 0.01 versus PA-treated group. (d) After treatment with BME and PA, cells were stained with DCFDA for 10 min. After washing twice with PBS, fluorescence intensity of DCFDA was determined by flow cytometry. Gated percentages are shown graphically. **p < 0.01 versus PA-treated group.

ER stress is one of the major mechanisms leading to apoptosis and PA is an inducer of ER stress in HepG2 cells (16–18). When ER stress occurs, BiP is activated to restore ER function. If ER function fails to recover, ER stress–specific markers are activated and highly expressed (19). Therefore, we investigated whether BME could decrease PA-induced ER stress markers in HepG2 cells by western blot. Our results revealed that levels of ER stress markers were significantly enhanced by PA treatment compared with those in the control (Fig. 2). Expression level of cleaved calnexin (C-calnexin) was also increased by PA treatment. However, expression level of C-calnexin was reduced after BME treatment. Expression level of BiP was slightly increased after BME treatment (Fig. 2a). Furthermore, PA-induced P-eIF2α and CHOP expression levels were decreased by BME in a dose-dependent manner (Fig. 2b). JNK and ROS are also involved in ER stress. Therefore, we examined the effect of BME on PA-induced JNK phosphorylation. As shown in Fig. 2c, BME suppressed PA-induced P-JNK and P-c-Jun levels. We also investigated the effect of BME on PA-induced ROS, a by-product of ER stress, in HepG2 cells. We found that BME inhibited PA-induced ROS production at concentrations ranging from 25 to 100 μg/mL (Fig. 2d), indicating that BME had antioxidant activity in HepG2 cells. Collectively, our data revealed that BME could decrease PA-induced ER stress and ROS production.

### BME inhibits HF-/HFr-induced hepatic injury

**Fig. 3:**
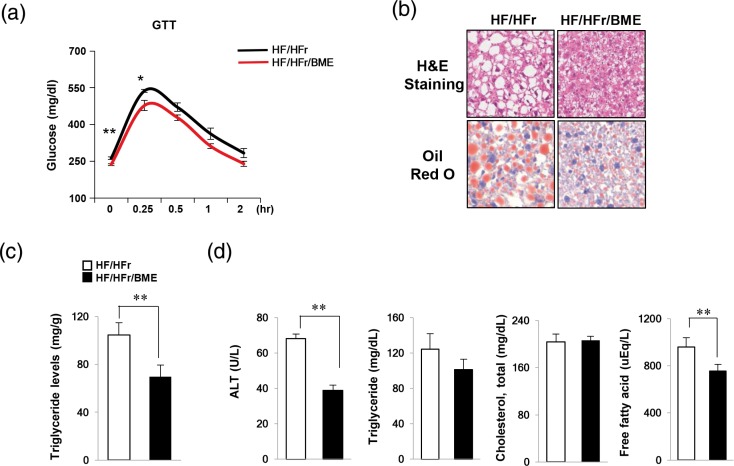
Protection effects of BME on fatty liver induced by HF/HFrD. (a) HF (containing 60% fat)/HFr (containing 30% fructose) water was administered to C57BL/6J mice for 7 weeks. Subsequently, the mice were randomly divided into two groups (*n* = 6 mice/group). Each group was orally administered either DMSO or BME (100 mg/kg) on every other day for 5 weeks under the HF/HFr diet. (a) After 5 weeks, GTT was then carried out by measuring glucose levels at 0, 0.25, 0.5, 1, and 2 h after glucose injection (1 g/kg). Livers were isolated from HF/HFr and HF-/HFr-/BME-fed mice. Data are presented as mean ± SEM (*n* = 6 per group). *p < 0.05, **p < 0.01 versus HF/HFr group. (b) Liver sections stained with hematoxylin & eosin and Oil Red O. (c) Liver TG was extracted using the Folch extraction method and TG level was then measured with a TG assay kit. (d) Levels of plasma ALT, TG, cholesterol, and FFA were measured using an autochemical analyzer. Data are presented as mean ± SEM (*n* = 6 per group). **p < 0.01 versus HF/HFr group (c, d).

Since we found that BME efficiently reduced apoptosis and ER stress in PA-treated HepG2 cells, we further examined the effect of BM on NAFLD *in vivo*. It has been reported that increased HF intake and HFr consumption might contribute to NAFLD pathogenesis in mice (20–22). We have also previously reported that high fat and high fructose diet fed mice showed severe NAFLD phenotype such as impaired glucose tolerance, increased lipid synthesis, and inflammation compared to control mice (23). In this study, C57BL/6J mice were fed 60% HFD and 30% fructose water (HF/HFr) for 7 weeks. Then, the mice were randomly divided into two groups: an HF/HFr group and an HF/HFr plus BME group (HF/HFr/BME). Each group was orally administered either DMSO or BME (100 mg/kg) on every other day for 5 weeks under the HF/HFr diet. During this period, the food intake was randomly measured three times and averaged. There was no difference in food intake between HF/HFr/BME group and HF/HFr group (Supplementary Fig. 2a), but BME significantly reduced body weight. Therefore, the effect of BME is not thought to have resulted from dietary control. We also measure the fat weight (Supplementary Fig. 2c). BME slightly reduced perirenal fat, but epididymal fat and subcutaneous fat did not change at all. Therefore, the effect of BME on body fat is considered to be negligible. As shown in Fig. 3a, GTT experiment revealed that HF/HFr/BME mice had significantly lower plasma glucose levels than HF/HFr mice. Interestingly, since BME significantly reduced liver weight (Supplementary Fig. 2c), we then performed H&E staining and Oil Red O staining to determine the effect of BME on the liver of mice. BME significantly prevented HF-/HFr- induced fat accumulation in the liver (Fig. 3b). Liver TG in HF/HFr was reduced by 30% after BME administration (Fig. 3c). Furthermore, the HF/HFr/BME group showed significantly lower levels of ALT, TG, and FFA compared to the HF/HFr group. However, cholesterol level did not differ significantly between the two groups (Fig. 3d). Taken together, these data suggest that BME can improve glucose tolerance, serum lipid levels, and fatty liver.

### BME reduces HF-/HFr-induced stress/inflammatory proteins and apoptosis

**Fig. 4:**
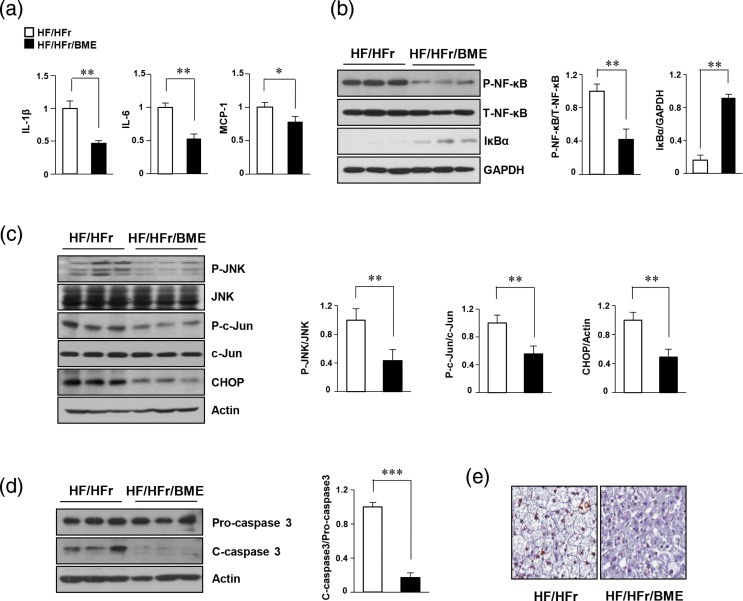
Beneficial effects of BME on inflammation and ER stress–mediated apoptosis in HF-/HFr-fed mice. Livers were isolated from HF/HFr and HF-/HFr-/BME-fed mice. (a) Hepatic mRNA was isolated from mice livers, and levels of interleukin IL-1β, IL-6, and MCP-1 mRNAs were quantified by real-time quantitative PCR. *p < 0.05, **p < 0.01 versus HF/HFr group. (b) Proteins isolated from mice livers. NF-κB protein levels were determined by western blot analysis. **p < 0.01 versus HF/HFr group. (c, d) Expression levels of P-JNK, P-c-Jun, CHOP, and C-caspase-3 were detected by western blot analysis. *p < 0.05, **p < 0.01, ***p < 0.001 versus HF/HFr group. (e) TUNEL staining of liver tissue sections showing DNA fragmentation.

We have previously reported that HF/HFr mice showed a higher inflammatory response than the control mice (23). Stress-/inflammation-related signals such as JNK and nuclear factor kappa B (NF-κB) are known to be involved in the induction liver injury caused by Western diet (24, 25). To determine whether BME could decrease HF-/HFr-induced activation of stress-/inflammation-related genes and apoptosis, levels of proteins and mRNAs extracted from the mice livers were examined by western blot and qPCR, respectively. Our results revealed that HF/HFr/BME livers showed significantly decreased inflammation-related genes, such as IL-1β, IL-6, and MCP-1 compared to HF/HFr livers (Fig. 4a). P-NF-κB levels also significantly reduced in BME-treated mice group (Fig. 4b). Based on results of our *in vitro* experiments, we next investigated the effect of BME on ER stress and apoptosis in HF/HFr and HF/HFr/BME groups of mice. As shown in Fig. 4c, a significant reduction in the expression level of ER stress markers like P-JNK, P-c-Jun, and CHOP was observed in HF/HFr/BME mice when compared to HF/HFr mice. The HF/HFr/BME mice showed lower C-caspase3 levels than HF/HFr mice (Fig. 4d). Terminal deoxynucleotidyl transferase dUTP nick and labeling (TUNEL) staining was then performed for liver tissues obtained from these two groups of mice. TUNEL-positive nuclei were observed in liver cells of HF/HFr mice. The number of TUNEL-positive cells was also higher in HF/HFr group than that in HF/HFr/BME group (Fig. 4e). These results clearly revealed that BME treatment can prevent ER stress-mediated apoptosis induced by inflammatory signals in HF/HFr mice.

## Discussion

Because BM has an effect on several diseases, BM has become a popular plant in the scientific community (10). Although effects of BM on numerous human diseases have been studied, studies on the effect of BM on NAFLD are insufficient. Therefore, this study focused on the effect of BME in ER stress–mediated apoptosis in PA-treated HepG2 cells as well as in an animal model of NAFLD.

NAFLD patients have increased delivery of FFA to the liver. Such elevated FFA can lead to serious diseases. It has been reported that PA belonging to FFA can increase ER stress–related protein CHOP and induce apoptosis by activating caspase-3 in HepG2 cells (15). Based on this report, we induced ER stress and apoptosis using PA in this study. The severity of disease progression in NAFLD patients and animal models is the result of hepatocyte cell death due to apoptosis. Fructose has been used to study its effect on hepatic steatosis, oxidative stress, and inflammation in hepatocyte apoptosis in NAFLD animal models (26, 27). The apoptotic signaling network involves a cascade of ER stress, ROS production, and inflammation intermixed with each other. In the pathogenesis of NAFLD, apoptosis is further accelerated by persistent and excessive ER stress (28). To maintain ER homeostasis and prevent apoptosis by ER stress, expression of chaperone BiP is increased to bind to misfolded proteins and activate the UPR response. Overexpression of BiP is known to protect saturated fatty-acid-induced apoptosis in HepG2 cells (29). If excessive ER stress persists, BiP will not be able to restore ER function. Therefore, other factors that cause apoptosis are activated. Calnexin is another ER chaperone known as an important marker in apoptosis triggered by ER stress. Proapoptotic function of calnexin can be regulated by BiP. It has been reported that overexpression of calnexin causes apoptotic death in *Schizosaccharomyces pombe* (30).

CHOP and caspase-3 are also prominent markers related to ER stress–mediated apoptosis. CHOP is activated by P-eIF2α. It has been reported that CHOP is a key marker of ER stress–mediated apoptosis. Reduction of apoptosis in ER stress has been demonstrated in CHOP(-/-)mice (31). In addition, Oyadomari and Mori have found that the reduction of CHOP in ER stress–mediated apoptosis is due to overexpression of BiP (32). Caspase-3 also promotes hepatocellular damage and pro-inflammatory signaling, leading to cell death. Caspase-3 inhibitors may be helpful for NAFLD progression (33, 34). Thus, targeting of BiP, calnexin, CHOP, and caspase-3 might be used as a potential strategy to treat NAFLD patients. Similar to previous reports, we also found that expression levels of CHOP, C-calnexin, and C-caspase-3 were efficiently decreased by BME, whereas BiP expression level was slightly increased in PA-treated HepG2 cells and animal model of NAFLD. These results suggest that BME can reduce apoptosis by inhibiting ER stress.

In fatty liver disease, excess supply of fatty acids and steatosis enhances ROS production due to ER dysfunction. ER stress signaling is associated with ROS formation and JNK activation. ROS produced by ER stress further promotes pro-inflammatory and pro-oncogenic signals (28). Oxidation of fatty acids activates inflammatory cytokines and produces ROS, leading to direct cell damage. Therefore, antioxidants are potentially therapeutic agents (29). In NAFLD, NF-κB and JNK are known to regulate metabolism through cell survival, inflammation, and apoptosis (1). In mouse models, it has been shown that HFDs and obesity can activate hepatic NF-κB which causes hepatic inflammation and increases the levels of IL-6, IL-1β, and TNF-α. These cytokines may also further increase NF-κB activation via a feed-forward mechanism (35). Interestingly, our results revealed that BME reduced ROS formation and levels of P-JNK and P-c-Jun. Furthermore, we found that NF-κB protein level was decreased and mRNA levels of IL-6, IL-1β, and MCP-1 were significantly lower in HF/HFr/BME mice than those in HF/HFr mice. Thus, BME might be able to alleviate PA-induced apoptosis by modulating ER stress–JNK signaling

HFD intake promotes hepatic steatosis and apoptosis. Guo et al. have reported that hepatic injury will occur when there is a lack of fat degradation or storage capacity due to excessive intake of fat and excess fatty acids in the liver. TG, a hallmark of NAFLD, is a major form of fat that accepts energy metabolism. It is accumulated in hepatocytes, causing steatosis. In mice fed with HFD, apoptosis is observed along with increased levels of ALT, cholesterol, glucose, and hepatocyte TG (36). It is well established that ALT levels are correlated with NAFLD severity and that reduction in serum ALT level is correlated with improvement of liver steatosis and inflammation (37). As expected, BME significantly reduced levels of glucose, TG, ALT, and FFA in HF/HFr mice sera. However, cholesterol levels were unchanged after BME treatment. Further studies are needed to clarify why BME could not affect cholesterol level.

In conclusion, this study demonstrated that BME could inhibit apoptosis by reducing ER stress in PA-treated HepG2 cells and an animal model of NAFLD. Our results suggest that BME might be useful for treating lifestyle-related diseases such as NAFLD.

## Supplementary Material

Bitter melon extract ameliorates palmitate-induced apoptosis via inhibition of endoplasmic reticulum stress in HepG2 cells and high-fat/high-fructose-diet-induced fatty liver

Bitter melon extract ameliorates palmitate-induced apoptosis via inhibition of endoplasmic reticulum stress in HepG2 cells and high-fat/high-fructose-diet-induced fatty liver
