# Supplementary figures and images for: Bitter melon extract ameliorates palmitate-induced apoptosis via inhibition of endoplasmic reticulum stress in HepG2 cells and high-fat/high-fructose-diet-induced fatty liver
Source: Food Nutr Res. 2018 Mar 22;62:10.29219/fnr.v62.1319. doi: 10.29219/fnr.v62.1319 (PMC5883859; doi:10.29219/fnr.v62.1319)

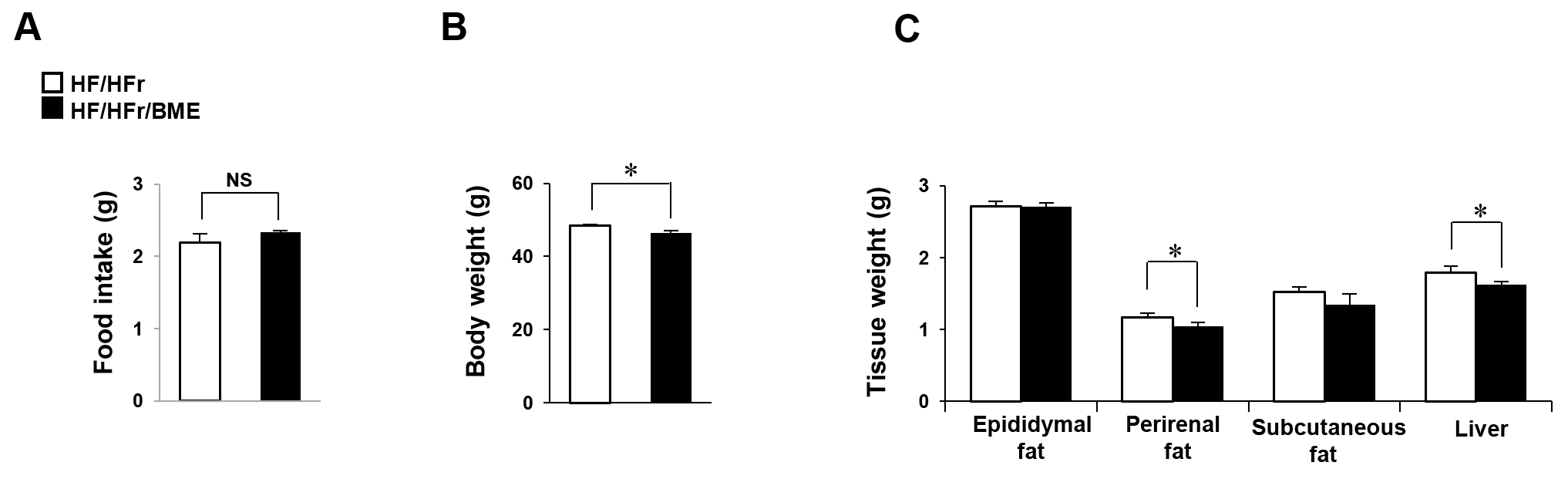

Supplement: Bitter melon extract ameliorates palmitate-induced apoptosis via inhibition of endoplasmic reticulum stress in HepG2 cells and high-fat/high-fructose-diet-induced fatty liver [file FNR-62-1319-s001.tif]

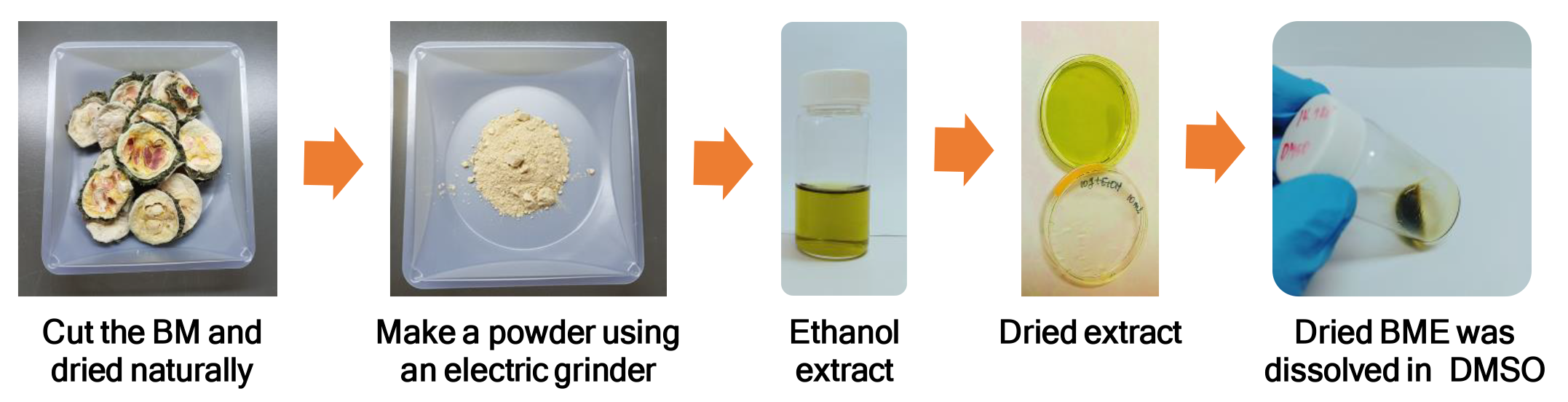

Supplement: Bitter melon extract ameliorates palmitate-induced apoptosis via inhibition of endoplasmic reticulum stress in HepG2 cells and high-fat/high-fructose-diet-induced fatty liver [file FNR-62-1319-s002.tif]
